# Acute Lower Leg Compartment Syndrome: A Rare Complication following CABG

**DOI:** 10.1155/2016/5268174

**Published:** 2016-07-21

**Authors:** Muazzam Tahir, Sean Galvin

**Affiliations:** Wellington Regional Hospital, Capital and Coast District Health Board, Wellington 6021, New Zealand

## Abstract

Compartment syndrome of lower legs following coronary artery bypass grafting is a rare complication which results from a combination of local and systemic factors. Early recognition is vital for good outcome. The case discussed describes this rare complication of CABG resulting in long term disability.

## 1. Introduction

Lower limb compartment syndrome is a rare complication following coronary artery bypass grafting [1–6]. It is a limb threatening surgical emergency [1, 2]; clinical examination and a high index of clinical suspicion allow for timely diagnosis and management of this condition [1] without long term disability.

## 2. Case Report

A 70-year-old man with history of unstable angina, hypertension, and impaired glucose tolerance was referred for coronary artery bypass grafting. Preoperative coronary angiogram showed double vessel disease with 90% ostial left anterior descending stenosis and 75% stenosis of the left circumflex bifurcation. Echocardiogram, preoperatively, showed left ventricular ejection fraction of 55% with preserved LV function. CABG was performed with left internal thoracic artery to left anterior descending artery and saphenous vein to the second obtuse marginal artery. The vein was harvested from the right lower leg. Crepe bandage with minimal compression was applied to the leg after surgery. There were no intraoperative complications.

Postoperatively he was successfully extubated and was transferred to ward on day 1 postoperatively from intensive care unit. He developed fast atrial fibrillation while on ward, managed with intravenous followed by oral amiodarone. The atrial fibrillation lasted for a few hours on day 2 postoperatively. On day three postoperatively, he complained of severe pain in right leg. On assessment the right leg was tense, tender, and swollen with minimal pain on passive flexion. Power in the foot was 1/5 on dorsiflexion and 5/5 on plantar flexion with loss of sensations over the dorsum of foot. He had foot drop on clinical exam with steppage gait. Foot pulses were not palpable with capillary refill time of more than 4 seconds. Nothing abnormal was found on neurovascular exam on the opposite side.

Doppler ultrasound scan of right leg excluded deep venous thrombosis. CTA lower limbs showed bilateral diffuse anterior tibial artery disease with no evidence of embolus (preoperative history and clinical examination were negative for peripheral vascular disease). Compartment pressures were measured in right leg which were elevated and he was diagnosed with the anterolateral compartment syndrome. Urgent fasciotomy was done. Muscles in the anterior compartment (tibialis anterior and external hallucis longus) were pale and were not contracting to diathermy stimulation. The wound was left open and a delayed closure was done by which time the muscles appeared to be pink. Pain resolved markedly after surgery; however, there was residual foot drop with impaired sensation over the dorsum of the foot. Once comfortable, he was transferred to the rehabilitation ward.

## 3. Discussion

Lower limb compartment syndrome following venous harvesting for CABG is rare [1–6] and multifactorial [1]. Cardiopulmonary bypass leads to hypotensive periods which can reduce perfusion to lower limbs [1, 7, 8] compounded by the peripheral vascular disease if present. Restoration of circulation following CPB can lead to reperfusion injury [4, 5]. Extracorporeal circulation results in release of proinflammatory cytokines leading to increased capillary permeability with accumulation of interstitial fluid and cellular swelling [8] which also contributes to pathogenesis [4, 5]. All these factors can lead to an increase in interstitial fluid and venous congestion with the initiation of a vicious circle resulting in reduced blood flow and compartment syndrome [1]. This can be further compounded by tight application of elastic bandage and inflammation with tissue oedema from surgical trauma [4, 5], explaining the frequent occurrence of compartment syndrome in donor limb [4, 5].

Lower limb compartment syndrome can be seen in both on and off pump CABGs [1, 6]. This condition which is commonly seen in vein donor leg [1, 6] can happen in the absence of peripheral vascular disease [3, 4]. This suggests that local factors, including inflammation and tissue oedema from surgical trauma and venous stasis from application of elastic bandages, are as important as systemic factors (hypoperfusion, reperfusion injury, and release of cytokines during the extracorporeal circulation) [1, 6]. Hence, care should be taken to avoid local injury [1].

In our case, CTA did not show acute limb embolus secondary to atrial fibrillation which is itself an independent and important risk factor for acute limb ischemia.

Lower limb compartment syndrome following CABG is a rare complication. A high index of clinical suspicion guided by clinical examination and measurement of the compartment pressure helps in diagnosing this limb threatening condition and fasciotomy should be considered.
